# Anthropometric measurements can identify small for gestational age newborns: a cohort study in rural Tanzania

**DOI:** 10.1186/s12887-019-1500-0

**Published:** 2019-04-23

**Authors:** Cecilie Bøge Paulsen, Birgitte Bruun Nielsen, Omari Abdul Msemo, Sofie Lykke Møller, Josephine Roth Ekmann, Thor Grundtvig Theander, Ib Christian Bygbjerg, John Peter Andrea Lusingu, Daniel Thomas Remias Minja, Christentze Schmiegelow

**Affiliations:** 1 Centre for Medical Parasitology, Department of Immunology and Microbiology, University of Copenhagen, Blegdamsvej 3B, Building 07-11-56, 2200 Copenhagen, Denmark; 2 Department of Obstetrics and Gynecology, Aarhus University Hospital, Aarhus, Denmark; 3 National Institute for Medical Research, Tanga Centre, Tanga, Tanzania; 4 Section of Global Health, Department of Public Health, University of Copenhagen, Copenhagen, Denmark

**Keywords:** Small for gestational age, Foot length, Chest circumference, Mid upper arm circumference, Gestational age, Africa, Positive and negative predictive value

## Abstract

**Background:**

Small-for-gestational-age (SGA) is associated with increased neonatal mortality and morbidity. In low and middle income countries an accurate gestational age is often not known, making the identification of SGA newborns difficult. Measuring foot length, chest circumference and mid upper arm circumference (MUAC) of the newborn have previously been shown to be reasonable methods for detecting low birth weight (< 2500 g) and prematurity (gestational age <  37 weeks). The aim of this study was to investigate if the three anthropometric measurements could also correctly identify SGA newborns.

**Methods:**

In the current study from a rural area of northeastern Tanzania, 376 live newborns had foot length, chest circumference, and MUAC measured within 24 h of birth. Gestational age was estimated by transabdominal ultrasound in early pregnancy and SGA was diagnosed using a sex-specific weight reference chart previously developed in the study area. Receiver operating characteristic curves were generated for each of the anthropometric measurements and the area under the curve (AUC) compared. Operational cutoffs for foot length, chest circumference, and MUAC were defined while balancing as high as possible sensitivity and specificity for identifying SGA. Positive and negative predictive values (PPV and NPV) were then calculated.

**Results:**

Of the 376 newborns, 68 (18.4%) were SGA. The AUC for detecting SGA was 0.78 for foot length, 0.88 for chest circumference, and 0.85 for MUAC. Operational cut-offs to detect SGA newborns were defined as ≤7.7 cm for foot length, ≤31.6 cm for chest circumference and ≤ 10.1 cm for MUAC. Foot length had 74% sensitivity, 69% specificity, PPV of 0.35 and NPV of 0.92 for identifying SGA. Chest circumference had 79% sensitivity, 81% specificity, PPV of 0.49 and NPV of 0.95 for identifying SGA. Finally, MUAC had 76% sensitivity, 77% specificity, PPV of 0.43 and NPV of 0.94 for identifying SGA.

**Conclusion:**

In a setting with limited availability of an accurate gestational age, all three methods had a high NPV and could be used to rule out the newborn as being SGA. Overall, chest circumference was the best method to identify SGA newborns, whereas foot length and MUAC had lower detection ability.

**Trial registration:**

Clinicaltrials.gov (NCT02191683). Registered 2 July 2014.

**Electronic supplementary material:**

The online version of this article (10.1186/s12887-019-1500-0) contains supplementary material, which is available to authorized users.

## Background

Sub-Saharan Africa has one of the highest neonatal mortality rates (death of a newborn per 1000 live births) in the world [1], and specifically in Tanzania the neonatal mortality rate is as high as 22.2 per 1000 live births [2]. A major risk factor for neonatal mortality is intrauterine growth restriction (IUGR) [3]. IUGR is difficult to diagnose, requiring a valid estimate of gestational age (GA), repeated fetal weight measurements in order to observe waning of fetal growth as well as Doppler flow measurement to identify poor placental function [4]. This is often problematic in low and middle income countries due to delayed and infrequent access to antenatal care as well as limited access of ultrasound examination [5, 6]. As surrogate markers of IUGR, small for gestational age (SGA) (a GA adjusted weight below a specific percentile on a reference weight chart) and low birth weight (LBW) (birth weight < 2500 g) are therefore used. Low birth weight encompasses both preterm and IUGR newborns and SGA is a better indicator of IUGR as GA is taken into account [5, 7]. It was estimated that 23 million children were born SGA in low and middle income countries in 2012 [8]. SGA is associated with an almost 2-fold increased risk of neonatal mortality and > 20% of neonatal deaths might be attributed to SGA [8]. Identifying SGA newborns and initiating proper care could therefore have tremendous health benefits. Simple interventions, including skin-to-skin contact to prevent hypothermia, early and frequent breastfeeding, and prevention as well as early management of infections have been shown to reduce neonatal mortality if targeting SGA newborns [7, 9–12].

In resource poor settings where estimation of an accurate GA is often difficult, alternative methods of identifying SGA newborns are warranted. Previous studies have shown foot length, chest circumference and mid upper arm circumference (MUAC) to be acceptable tools for identifying LBW [9–11, 13–16] and premature (GA <  37 weeks) newborns [9, 11, 13, 14]. In these studies, ultrasound in early pregnancy for GA estimation was not available, nor was a representative weight chart; both are key components when diagnosing SGA [4]. We conducted a preconceptional-pregnancy cohort study in Tanzania investigating the effect of anemia on fetal and placental development. GA was estimated using ultrasound in early pregnancy, the newborn was assessed within 24 h of birth, and a representative sex-specific weight reference chart was available for the study area [17]. The aim of the presented sub-study was to evaluate if foot length, chest circumference or MUAC could be used to diagnose SGA at birth.

## Methods

### Study design

**Fig. 1:**
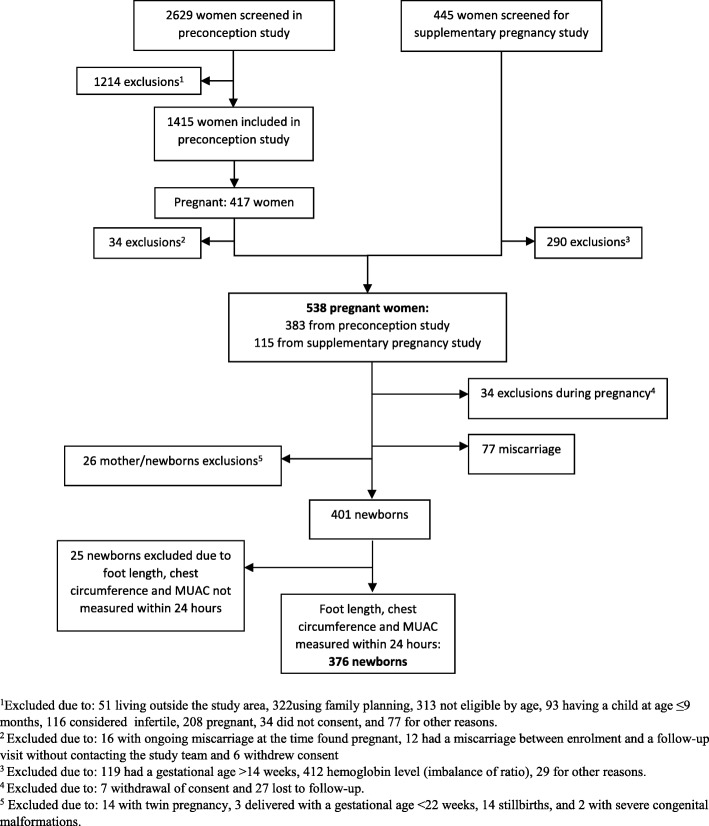
Flow chart of the study population

The study was part of the FOETALforNCD study carried out in Korogwe and Handeni Districts, northeastern Tanzania, a predominantly rural area with Korogwe District Hospital as the main health facility. Women were screened for enrolment between July 2014 and March 2016, and follow-up was completed in December 2016 (Fig. 1).

The FOETALforNCD study consisted of two cohorts; a preconceptional cohort that enrolled women before pregnancy who were followed throughout pregnancy if they conceived, and a pregnancy cohort that enrolled pregnant women with a GA of ≤14 weeks. In the preconceptional cohort, the inclusion criteria were age between 18 and 40 years, no current use of modern family planning except condoms, a negative pregnancy test, not having a baby under 9 months of age, not having tried to conceive unsuccessfully for > 2 years consecutively and having consented to attend all the antenatal care and giving birth at Korogwe District Hospital if they conceived during the study period. In the pregnancy cohort women were included if having a GA ≤14 weeks and based on a 1:1 distribution of maternal anemia (hemoglobin < 11 g/dL) and no maternal anemia (hemoglobin≥11 g/dL).

### Data collection

Pregnancy was confirmed with a urine human Chorionic Gonadotropin test (One step pregnancy test strip; Vista Care Company, Shandong, China with a sensitivity of 25 mlU/ml human Chorionic Gonadotropin) followed by transabdominal ultrasound for GA estimation (Sonosite TITAN and Turbo, US High resolution, Bothell, WA, USA) using crown-rump-length in the 1st trimester [18] and head circumference in the 2nd trimester [19].

Data collection throughout pregnancy was similar in the two cohorts and was conducted in the local language Swahili and then documented into case report forms in English. At enrolment data on demographics, socio-economic status, and medical and obstetric history were collected. After enrolment in the pregnancy part of the study scheduled visits were conducted at GA of 11–14, 20–22, 26–28, 32–34 and 37–39 weeks. At every contact, a medical examination was done including blood pressure (R-champion N, Rudolf Riester, Jungingen, Germany), anthropometric measurements, urine dipstick (Combiscreen 7, Alere), hemoglobin level (venous blood using Sysmex KX-21 N hematological analyzer, Sysmex Corporation Kobe, Japan) and malaria screening (using malaria rapid diagnostic tests (ParaHIT, Span Diagnostics, Gujarat India or CareStart® Access Bio NJ, USA), blood smear for malaria microscopy, and dried blood spot for malaria species diagnostic PCR). Hypertensive disorders were defined as systolic blood pressure ≥ 140 mmHg and/or diastolic blood pressure ≥ 90 mmHg observed on at least two measurements taken > 4 h apart or systolic blood pressure ≥ 160 mmHg and/or diastolic blood pressure ≥ 110 mmHg observed at least once. Preeclampsia was defined as hypertension and proteinuria on at least two occasions after a GA of 20 weeks. Anthropometric measurements included height in centimeters (cm) (only at enrolment) (Stadiometer, SECA GmbH & Co. KG, Hamburg, Germany), and weight without outer garment and shoes recorded to nearest 0.1 kg (Digital weighing scale, SECA GmbH & Co. KG, Hamburg, Germany) [20]. Body mass index was calculated by dividing the body weight with the square of the height.

At birth a thorough anthropometric examination of the newborns was performed within 24 h. Birth weight was measured on a nude newborn using a digital baby weighing scale (M107600, ADE, Germany) and noted in grams (g) to the nearest 5 g [21]. The length of the newborn was measured from the vertex to the heel of the right foot using an infantometer (Baby Infantometer 417, SECA GmbH & Co. KG, Hamburg, Germany) [21]. The foot length was measured from the heel to the tip of the longest toe on the right foot using a hard transparent plastic ruler and noted in cm [9, 11, 13]. Chest circumference and MUAC were measured with a flexible non-stretchable tape measure. Chest circumference was measured to the nearest 0.1 cm on a calm baby (mid-expiration) by circling the chest at the level of the nipples [22]. The MUAC was measured to the nearest 0.1 cm at the right upper arm mid-point halfway between the acromion of the scapula and the olecranon of the ulna [20]. All measurements were performed twice. If the difference between two measurements exceeded 50 g for birthweight, 7 mm for length, 5 mm for chest circumference, and 2 mm for foot length or MUAC, a third measurement was done and the two measurements closest to each other were documented. APGAR score, the appearance of amniotic fluid and any congenital malformations were documented as well. A sex-specific weight reference chart previously developed in the same study area [17] was used to define SGA as a birth weight below the 10th percentile. LBW was defined as birth weight < 2500 g [7] and preterm as a GA < 37 weeks [7]. In addition, the premature newborns were defined as extremely preterm (< 28 weeks), very preterm (28 to < 32 weeks) and moderate to late preterm (32 to < 37 weeks) [23].

### Ethical considerations

Ethical approval was granted by the Medical Research Coordinating Committee of the National Institute for Medical Research in Tanzania (reference number NIMR/HQ/R.8a/Vol. IX/1717). All study participants gave written informed consent (or thumbprints from illiterate women) before enrolment. All study participants were given unique identification numbers to ensure anonymity, and only authorized personnel had access to the data. All project activities were conducted in accordance with Good Clinical Practice and the Declaration of Helsinki. Participants were assisted by the project in obtaining the best local medical care available if a disease was diagnosed during the study period.

### Statistical methods

All data were checked for consistency, double entered, and validated using Microsoft Office Access 2007 database (Microsoft Corporation, Redmond USA). All analyses were performed using R version 3.4.3 (2017-11-30), Copyright (C) 2017 The R Foundation for Statistical Computing.

Only singleton, liveborn newborns at a GA > 22 weeks without severe congenital malformations and with neonatal examination done within 24 h were included in the analysis. The study population was described with mean and standard deviation for parametric continuous data, median and interquartile range for non-parametric data, and proportion for categorical data (number (%)). Finally, for GA at delivery and birth weight the range with minimum and maximum values were reported and the 2.5th and 97.5th percentiles for birth weight were calculated. The association between foot length, chest circumference and MUAC versus birth weight and GA at delivery was calculated with Pearson and Spearman correlations, respectively. Student’s t-test was used for comparison of mean foot length, chest circumference and MUAC for SGA, LBW, and preterm newborns compared to normal weight and term newborns as well as for comparison of mean foot length, chest circumference and MUAC among male and female newborns.

Receiver operating characteristics (ROC) analysis was conducted separately for each anthropometric measurement and the area under the curve (AUC) calculated to investigate which measurement best predicted SGA, LBW and prematurity, respectively. For sensitivity analyses, ROC analysis only including newborns with GA estimated ≤14 weeks and ROC analysis stratified by the sex of the newborn were performed. Operational cut-offs for foot length, chest circumference and MUAC were selected based on obtaining the highest sensitivity and specificity for identifying SGA, and positive predictive value (PPV) and negative predictive value (NPV) for SGA were then calculated. Using the same cut-offs; sensitivity, specificity, PPV, and NPV were also calculated for LBW and prematurity. For comparison, operational cut-offs for foot length, chest circumference and MUAC were also selected based on obtaining the highest sensitivity and specificity for identifying either LBW or prematurity. The 95% confidence intervals for all the sensitivities, specificities, PPVs and NPVs were reported.

### Results

In total, 538 women were enrolled in the pregnancy part of the study, and hereof 401women gave birth to a live singleton newborn without severe congenital malformations. Among these, 376 newborns had foot length, chest circumference and MUAC measured within 24 h and were included in the analyses (Fig. 1). The median GA was 40 weeks + 0 days (Interquartile range 38 + 6 to 41 + 1, range 25 + 6 to 45 + 0) and the mean birth weight 3014 g (±486, range 860 to 4360, 2.5th to 97.5th percentiles 2061 to 3968). In total, 68 (18.4%) were born SGA, 39 (10.4%) were LBW, and 17 (4.5%) were born preterm. Among the preterm newborns, 15 (88.2%) were moderate to late preterm, 1 (5.9%) was very preterm and 1 (5.9%) was extremely preterm. The characteristics of the mothers and their newborns are shown in Table 1.

**Table 1:** Characteristics of the 376 women and their newborns

Characteristics	Mean ± SD^f^, median (interquartile range) or number (%)
Maternal age (years)^a^	26 (23.2–32.1)
Maternal Body Mass Index (kg/m^2^)^b^	
Underweight (< 18.5)	24 (6.5%)
Normal weight (18.5–24.9)	233 (63.0%)
Overweight (> 25)	113 (30.5%)
Gravidity	
≤ 2	106 (28.2%)
> 2	270 (71.8%)
Parity	
≤ 2	204 (54.3%)
> 2	172 (45.7%)
Malaria during pregnancy	144 (38.3%)
Hypertensive disorder during pregnancy	17(4.5%)
Gestational age at inclusion (weeks + days)^c^	8 + 6 (7 + 3–13 + 3)
Gestational age at delivery (weeks + days)	40 + 0 (38 + 6–41 + 1)
Female sex	193 (51.3%)
Caesarean section	25 (6.6%)
Birth weight (g)^d^	3014 ± 486
Length (cm)	48.3 ± 2.5
Foot length (cm)	7.8 ± 0.5
Chest circumference (cm)	32.4 ± 2.1
Mid upper arm circumference (cm)	10.5 ± 1.1
Small for gestational age (SGA)^e^	68 (18.4%)
Low birth weight (< 2500 g) (%)	39 (10.4%)
Preterm (< 37 weeks) (%)	17 (4.5%)

^a^371 women had age estimated at enrolment, ^b^370 women had body mass index estimated, ^c^316 (84.0%) women had gestational age estimated at ≤14 weeks, 54 (14.4%) women had gestational age estimated between 15 and 24 weeks and 6 (1.6%) had gestational age estimated at 25–28 weeks, ^d^375 had BW estimated, ^e^369 newborns were categorized as either SGA/non-SGA, ^f^*SD* Standard deviation

### Association between newborn foot length, chest circumference and MUAC with SGA, preterm delivery and LBW

**Fig. 2:**
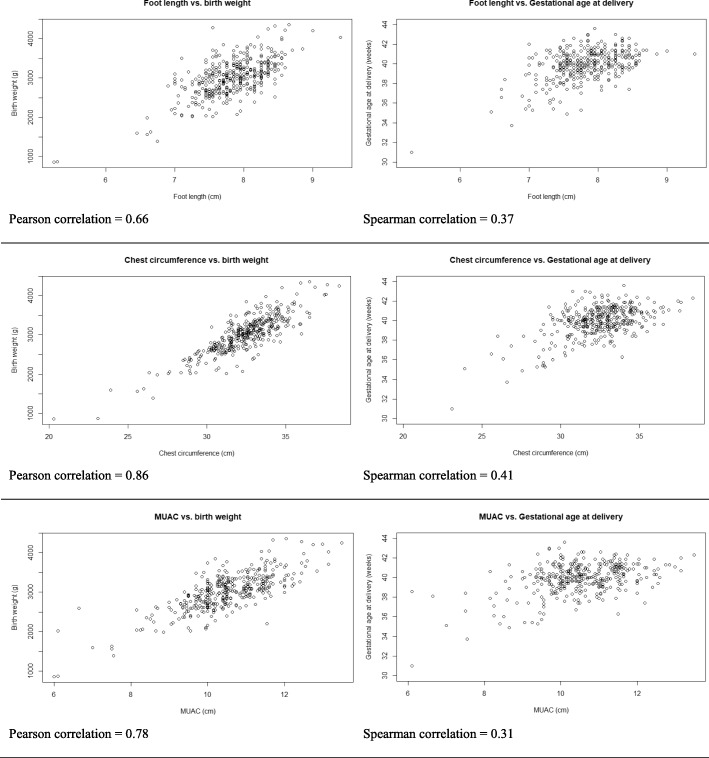
Association between foot length, chest circumference and MUAC vs. birth weight and gestational age at delivery

All three anthropometric measurements of the newborns correlated with birth weight and GA at delivery, showing the best correlation when using chest circumference (Pearson correlation for birth weight 0.86, Spearman correlation for GA 0.41) (Fig. 2). All three anthropometric measurements were also statistical significantly smaller among newborns born SGA, with LBW or preterm as compared to newborn with normal weight and born at term (Table 2). The anthropometric measurements were comparable for boys and girls except for foot length which was significantly shorter for girls (difference = 0.12 cm, *p*-value = 0.01, 95% confidence interval (CI) (0.02–0.21)) [See Additional file 1].

**Table 2:** Differences in foot length, chest circumference and mid-upper-arm-circumference among the 376 newborns

	Foot length (cm)		Chest circumference (cm)		MUAC^b^ (cm)	
	Mean ± SD	*p*-value	Mean ± SD	*p*-value	Mean ± SD	*p*-value
SGA^a^	7.5 ± 0.5	< 0.0001	30.2 ± 2.1	< 0.0001	9.4 ± 1.1	< 0.0001
Non -SGA	7.9 ± 0.4		32.9 ± 1.7		10.8 ± 0.9	
Low birth weight	7.2 ± 0.6	< 0.0001	28.8 ± 2.6	< 0.0001	8.9 ± 1.3	< 0.0001
Non-Low birth weight	7.9 ± 0.4		32.8 ± 1.6		10.7 ± 0.9	
Preterm	6.9 ± 0.7	< 0.0001	27.7 ± 3.2	< 0.0001	8.6 ± 1.4	< 0.0001
Term	7.9 ± 0.4		32.6 ± 1.7		10.6 ± 1.0	

^a^*SGA* Small for gestational age. ^b^*MUAC* Mid upper arm circumference

### ROC curve analysis

**Fig. 3:**
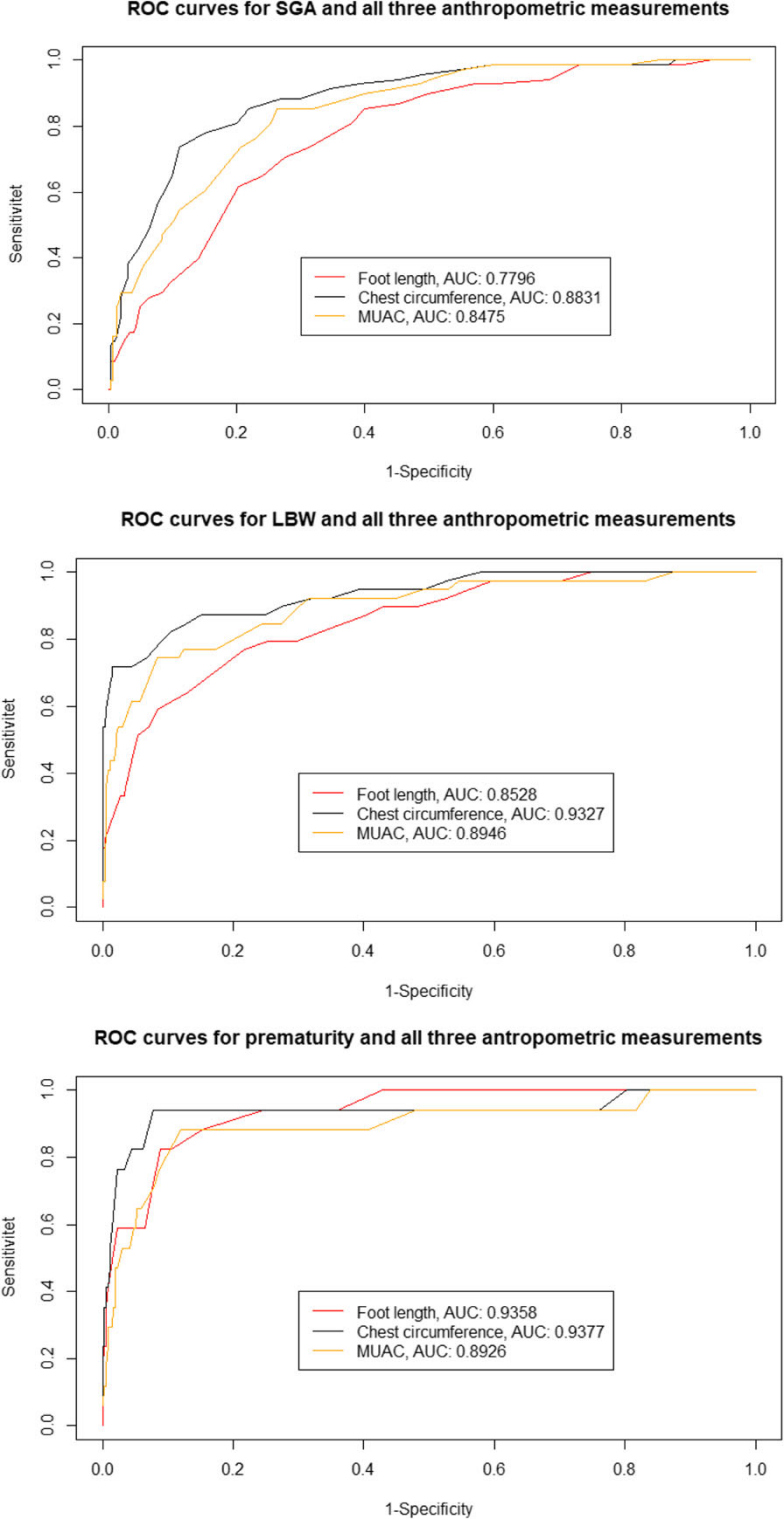
ROC curves for detecting SGA, low birth weight and prematurity, respectively using the three anthropometric measurements

ROC curve analyses were used to assess the three different anthropometric measurements’ ability to capture SGA, LBW and prematurity. Chest circumference had the highest AUC for all three outcomes. The highest observed AUC was for chest circumference detecting prematurity (0.94) while foot length detecting SGA had the lowest (0.78) (Fig. 3). A minority (60/376, 16%) of the women had GA estimated after 14 weeks of pregnancy. Sensitivity analysis only including newborns where GA was estimated ≤14 weeks yielded similar AUC for all three anthropometric measurements [See Additional file 2]. If stratifying by the sex of the newborn, AUC’s for chest circumference and MUAC were comparable for boys and girls, whereas the AUC for foot length was slightly higher for girls [See Additional file 3 and Additional file 4].

### Selection of anthropometric operational cut-offs

The sensitivity and specificity for detecting SGA was calculated for a range of different cut-offs of foot length, chest circumference and MUAC. The operational cut-offs yielding the highest combination of sensitivity and specificity (equivalent to the place on the ROC most to the top left) for detecting SGA newborns were defined as ≤7.7 cm for foot length (sensitivity at 74%, specificity at 69%), ≤31.6 cm for chest circumference (sensitivity at 79%, specificity at 81%) and ≤ 10.1 cm for MUAC (sensitivity at 76%, specificity at 77%) (Table 3). If stratified by sex of the newborn the operational cut-off for foot length for boys yielding the highest combination of sensitivity and specificity for detecting SGA was similar to the already defined operational cut-off. However, for girls the cut-off for foot length was slightly lower (≤ 7.6 cm) [See Additional file 5]. The other anthropometric measurements did not differ significantly for boys and girls.

**Table 3:** Sensitivity, specificity, PPV and NPV for operational anthropometric cut-offs together with the 95% confidence interval

		Sensitivity (%) (95% CI^e^)	Specificity (%) (95% CI)	PPV^f^ (95% CI)	NPV^g^ (95% CI)
SGA^a^	FL^b^ ≤ 7.7 cm	74 (61–83)	69 (63–74)	0.35 (0.27–0.43)	0.92 (0.88–0.95)
	CC^c^ ≤ 31.6 cm	79 (68–88)	81 (76–85)	0.49 (0.39–0.58)	0.95 (0.91–0.97)
	MUAC^d^ ≤ 10.1 cm	76 (65–86)	77 (72–82)	0.43 (0.34–0.52)	0.94 (0.90–0.96)
Low birth weight	FL ≤7.7 cm	82 (66–92)	67 (61–72)	0.22 (0.16–0.30)	0.97 (0.94–0.99)
	CC ≤ 31.6 cm	87 (73–96)	76 (71–81)	0.30 (0.22–0.39)	0.98 (0.96–0.99)
	MUAC ≤10.1 cm	85 (69–94)	73 (68–77)	0.26 (0.19–0.35)	0.98 (0.95–0.99)
Preterm	FL ≤ 7.7 cm	94 (71–100)	64 (59–69)	0.11 (0.06–0.17)	1.00 (0.98–1.00)
	CC ≤ 31.6 cm	94 (71–100)	72 (67–77)	0.14 (0.08–0.22)	1.00 (0.98–1.00)
	MUAC ≤10.1 cm	88 (64–99)	69 (64–74)	0.12 (0.07–0.19)	0.99 (0.97–1.00)

^a^*SGA* Small for gestational age, ^b^*FL* Foot length, ^c^*CC* Chest circumference, ^d^*MUAC* Mid upper arm circumference, ^e^*CI* Confidence interval, ^f^*PPV* Positive predictive value, ^g^*NPV* Negative predictive value

### Positive and negative predictive value and 95% confidence interval for the operational cut-offs

All three anthropometric measures show high NPVs (0.92–0.95) for identifying SGA, whereas the PPVs were below 0.50 for all (0.35–0.49). The best PPV and NPV were observed when using chest circumference (0.49 and 0.95, respectively) (Table 3). The main objective was to assess the ability of foot length, chest circumference and MUAC in identifying SGA. Since prematurity and LBW are closely related to SGA, sensitivity, specificity, PPV, and NPV for the same operational cutoffs, but using LBW or prematurity as outcome, were also calculated. As compared to when detecting SGA, sensitivity and NPV were higher for both preterm delivery and LBW for all three anthropometric measurements, whereas specificity and PPV were slightly lower (Table 3).

For comparison operational anthropometric cut-offs balancing the sensitivity and specificity for detecting LBW and preterm delivery instead of SGA were also calculated. Cut-offs for all three anthropometric measures were slightly smaller for both LBW and preterm delivery. For both outcomes specificities and PPVs increased, whereas sensitivities and NPV were either comparable or slightly decreased as compared to when using the operational cut-offs defined based on SGA (Table 4).

**Table 4:** Sensitivity and specificity of operational anthropometric cut-offs to identify Low birth weight and preterm

		Sensitivity (%)	Specificity (%)	PPV^a^	NPV^b^
		(95% CI)	(95% CI)	(95% CI)	(95% CI)
Low birth weight	FL^c^ ≤ 7.6 cm	79 (64–91)	75 (70–79)	0.27 (0.19–0.36)	0.97 (0.94–0.99)
	CC^d^ ≤ 31.2 cm	85 (69–94)	85 (81–89)	0.40 (0.30–0.52)	0.98 (0.96–0.99)
	MUAC^e^ ≤ 9.9 cm	77 (61–89)	83 (78–87)	0.34 (0.24–0.45)	0.97 (0.94–0.99)
Preterm	FL ≤ 7.45 cm	88 (64–99)	85 (81–88)	0.21 (0.13–0.33)	0.99 (0.98–1.00)
	CC ≤ 30.3 cm	94 (71–100)	92 (89–95)	0.36 (0.22–0.52)	1.00 (0.98–1.00)
	MUAC ≤9.6 cm	88 (64–99)	88 (84–91)	0.26 (0.15–0.39)	0.99 (0.98–1.00)

^a^ *PPV* Positive predictive value, ^b^ *NPV* Negative predictive value, ^c^*FL* Foot length, ^d^*CC* Chest circumference, ^e^*MUAC* Mid upper arm circumference

## Discussion

In a resource poor setting with high neonatal mortality rates and limited access to equipment for accurately estimating GA, there is a need for alternative methods to identify SGA newborns. We found that foot length, chest circumference and MUAC all correlated well with birth weight and GA and had reasonable sensitivity and specificity for the detection of SGA. Furthermore, high NPVs to detect SGA were observed for all three anthropometric measurements.

The characteristics of our newborns were similar to previous reports from Africa: birth weight of 3014 g and LBW rate of 10.4% as compared to birth weights of 2900–3050 g [9, 13, 15] and LBW rates of 12–15% [9, 10, 13]. The mean foot length, chest circumference and MUAC also corresponded well to the mean foot length [9, 10, 13], chest circumference [10] and MUAC [10] reported in studies from other parts of Tanzania and Uganda. Finally, prematurity rate was similar to the 4–8% reported from southern Tanzania [13], Uganda [9] and Nepal [11]. In line with other studies [10, 24] we found a statistically significant difference in anthropometric measurements between newborns born SGA, LBW and preterm as compared to normal weight and term newborns. For all three anthropometric measures, the AUCs for identifying SGA newborns were noticeably lower compared to the AUCs for identifying LBW and premature newborns. Previous studies also reported slightly higher sensitivities and specificities for detecting LBW or prematurity than we did for SGA [9–11, 13, 14, 22, 25]. We believe our measuring technique was adequate as the observed AUCs for predicting LBW and prematurity in our study were comparable to findings from previous studies [10, 14, 15]. Furthermore, if defining our operational cut-off based on LBW or prematurity as the outcome, sensitivity and specificity was similar to previous studies [9–11, 13, 14]. This could therefore suggest that SGA newborns might be a more difficult group to identify by anthropometric measurements.

The operational cut-offs at ≤7.7 cm, ≤31.6 cm and ≤ 10.1 cm for foot length, chest circumference and MUAC, respectively, were in line with previous studies reporting cut-offs of 7.0–8.0 cm for foot length [9–11, 13, 14, 22, 25], 29.8–31.5 cm for chest circumference [10, 14, 15, 22, 25] and 8.9–10.1 cm for MUAC [10, 14, 15, 25] for the identification of LBW. When identifying preterm newborns, the cut-off for foot length has been reported as 7.5–8.0 cm which is also similar to our cut-off [9, 13]. In general, the studies conducted in Asia [11, 14, 22] as compared to Africa [9, 10, 13, 15] report shorter operational cut-offs for identifying LBW and prematurity [11, 14, 22]. This could implicate the need for region specific cut-offs. Of note, however, is that the methodology for selecting the cut-offs varies from study to study. The most frequently used method, which we also applied in our study, was to determine the cut-off as the one yielding the highest average of sensitivity and specificity to detect an outcome [9–11, 22]. However, other studies used the highest [(sensitivity + specificity)/2] ratio [15], the point with the highest sensitivity and specificity such that the sensitivity was at least 0.8 [14] or linear regression to obtain an optimal cut-off [25]. This could explain some of the difference in the cut-offs reported. Finally, differences between male and female newborns were limited. However, foot length was slightly shorter for girls suggesting that sex-specific cut-offs could be considered for foot length.

Sensitivity was 74–79% and specificity 69–81% for the operational cut-offs for identifying SGA newborns. Furthermore, all methods yielded high NPVs (0.92–0.95), whereas PPVs were below 0.50. Especially the use of chest circumferences gave promising results showing the highest values of sensitivity, specificity, PPV and NPV for detecting SGA. Among newborns with a chest circumference > 31.6 cm, 95% would be correctly categorized as non-SGA. This suggests that chest circumference is a valid tool to exclude SGA. On the contrary, the low PPV meant that among newborns with a chest circumference ≤ 31.6 cm more than half of the newborns would wrongly be classified as SGA. In the context of the high neonatal mortality rate in the Sub-Saharan Africa, a high NPV is of greater importance than a high PPV. The consequences of being misdiagnosed as SGA is limited as the interventions will only promote extra care. However, the consequences of misclassifying an SGA newborn as non-SGA could be of significant importance, as SGA is associated with an increased risk of mortality [8].

Although all anthropometric measurements are simple and requires little training to master, the clinical project staff in FOETALforNCD received repetitive training and supervision in an attempt to avoid measurement errors. Furthermore, the operational anthropometric cut-offs to identify SGA newborns were not known during data collection. In a setting with a limited public health care system, it would be preferable with one anthropometric cut-off for identification of the at-risk newborns in need of extra care, avoiding a scenario with multiple anthropometric cut-offs identifying a variety of conditions. This might only induce confusion among health care professionals and unnecessary amount of time is spent. It has previously been argued that foot length is preferable as the exposure to hypothermia is limited [22]. However, foot length performed the poorest with the lowest AUC, PPV and NPV. Based on our results we would recommend chest circumference with the 31.6 cm cut-off defined when using SGA as an outcome. The NPV is ≥95% for all outcomes, and NPV for LBW and prematurity did not improve if using the cut-offs defined with either as an outcome instead.

The strength of this study was the accurate GA estimation using ultrasound in early pregnancy as well as the availability of a representative weight reference chart generated in a previous project conducted in the same area [17]. Birth weight is often possible to measure even in rural settings. GA is rarely available leading to difficulties in correctly diagnosing prematurity and SGA. Our study support that using newborns anthropometrics can be an alternative diagnostic method to identify the newborns most at risk in settings with limited access to GA. Other methods, last menstrual period and symphysio-pubis fundal height, have been shown to underestimate GA, emphasizing the importance of using ultrasound in this estimation [26]. The above enabled us, as the first ones, to investigate if anthropometrics of the newborn could be used to capture SGA.

Some of our women did not have GA estimated until the 2nd trimester. Moore et al. showed that the GA estimated by head circumference underestimated GA by 0.39 weeks, particularly if the ultrasound scan was performed after 24 weeks [27]. The fact that 54 (14.4%) women had GA estimated between 15 and 24 weeks and 6 (1.6%) had GA estimated at 25–28 weeks could therefore have artificially lowered the prevalence of SGA and prematurity. However, when only including newborns with GA estimated within the first 14 weeks of pregnancy similar AUC was obtained and we therefore do not believe the later GA estimation for some of the women has influenced the results substantially.

Our findings are based on a population where 95% of the birth weights were between 2061 g - 3968 g. The diagnostic ability of newborn anthropometry to correctly identify LBW and prematurity found in our study was comparable to what has been observed in studies from Africa with birth weight ranges similar to ours [9, 13]. Diagnostic ability might vary depending on newborn size. Therefore, the findings in our as well as previous studies might not be applicable in a population with a considerable different birthweight distribution.

The findings of our study should be confirmed in larger studies. Furthermore, there might be differences in the size of newborns with different ethnic and geographic origin [28, 29]. It would therefore be beneficially if future studies on the diagnostic ability of the of newborn anthropometrics to identify SGA were conducted in populations with both similar as well as different maternal and newborn characteristics as compared to ours.

## Conclusion

In a setting with limited availability of an accurate GA, foot length, chest circumference and MUAC had high NPV and could be used to rule out the newborn as being SGA. Overall, chest circumference was the best method to identify SGA newborns, whereas foot length and MUAC have poorer detection ability. When identifying at-risk newborns extra care in the shape of simple interventions can be initiated with the potential of reducing neonatal mortality.

## Additional files

Additional file 1: **Table S1.** Differences in foot length, chest circumference and mid-upper-arm-circumference (MUAC) among the sexes (PDF 62 kb)

Additional file 2: **Figure S1.** ROC curves for SGA and all three anthropometric measurements – only including newborns with gestational age estimated within 14 weeks (PDF 36 kb)

Additional file 3: **Figure S2.** ROC curves for SGA and all three anthropometric measurements – only including boys (PDF 19 kb)

Additional file 4: **Figure S3.** ROC curves for SGA and all three anthropometric measurements – only including girls (PDF 34 kb)

Additional file 5: **Table S2.** Foot length cut-off identifying small for gestational age when stratified by sex together with the 95% confidence interval (CI) (PDF 57 kb)

## Abbreviations

AUC: Area under the curve
cm: Centimeters
g: Grams
GA: Gestational age
IUGR: Intra uterine growth restriction
LBW: Low birth weight
MUAC: Mid upper arm circumference
NPV: Negative predictive value
PPV: Positive predictive value
ROC: Receiver operating characteristics
SGA: Small for gestational age
