# Supplementary material for: Anthropometric measurements can identify small for gestational age newborns: a cohort study in rural Tanzania
Source: BMC Pediatr. 2019 Apr 23;19:120. doi: 10.1186/s12887-019-1500-0 (PMC6477730; doi:10.1186/s12887-019-1500-0)
Supplement: Supplementary file 1 — Table S1. Differences in foot length, chest circumference and mid-upper-arm-circumference (MUAC) among the sexes (PDF 62 kb) [file 12887_2019_1500_MOESM1_ESM.pdf]

Supplementary Table 1. Differences in foot length, chest circumference and mid-upper-arm-circumference (MUAC) among the sexes.

	Foot length (cm)		Chest circumference (cm)		MUAC (cm)	
	Mean ± SD	p-value	Mean ± SD	p-value	Mean ± SD	p-value
Boys	7.9 ± 0.5	0.0139	32.6 ± 2.1	0.1781	10.6 ± 1.0	0.2159
Girls	7.8 ± 0.4		32.3 ± 2.1		10.4 ± 1.1	
