# Supplementary figures and images for: Anthropometric measurements can identify small for gestational age newborns: a cohort study in rural Tanzania
Source: BMC Pediatr. 2019 Apr 23;19:120. doi: 10.1186/s12887-019-1500-0 (PMC6477730; doi:10.1186/s12887-019-1500-0)

Supplementary Figure 1.

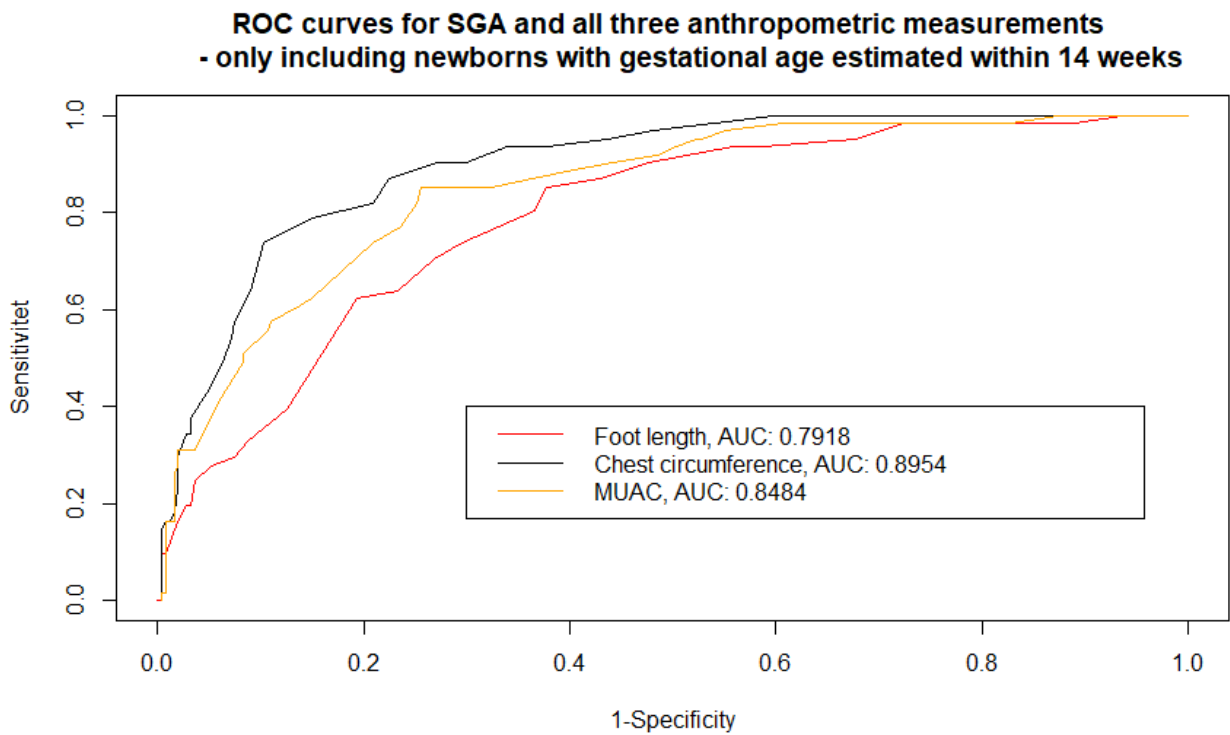

Supplement: Supplementary file 2 — Figure S1. ROC curves for SGA and all three anthropometric measurements – only including newborns with gestational age estimated within 14 weeks (PDF 36 kb) [file 12887_2019_1500_MOESM2_ESM.pdf]

Supplementary Figure 2.

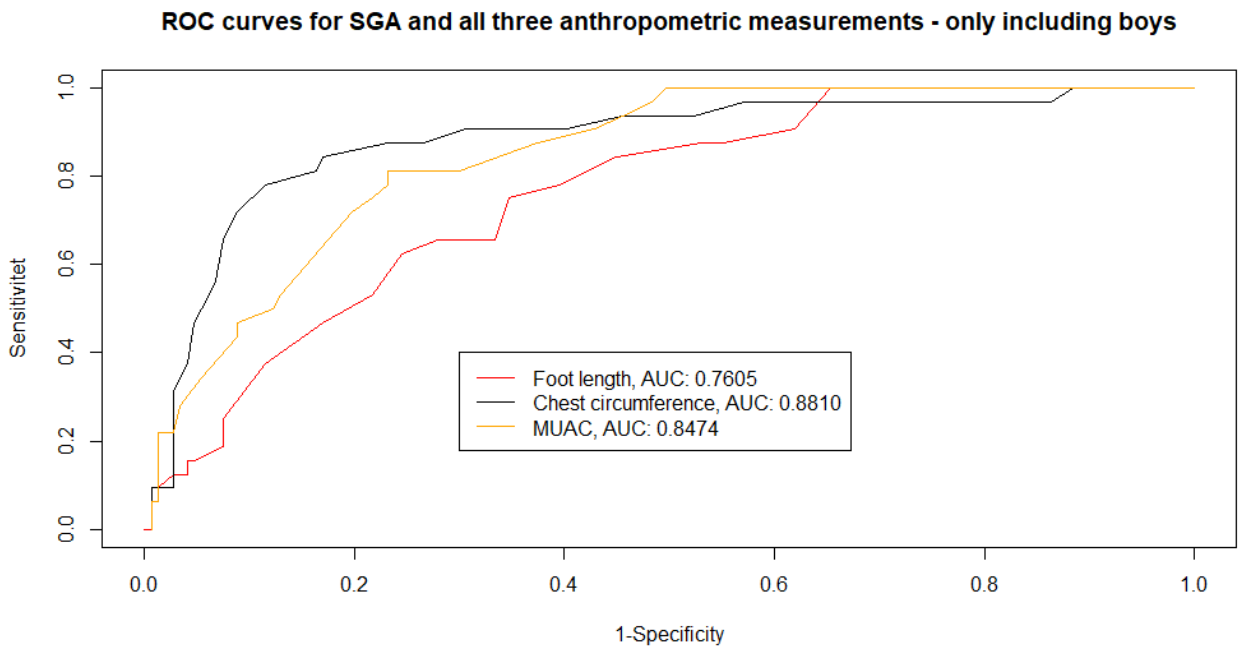

Supplement: Supplementary file 3 — Figure S2. ROC curves for SGA and all three anthropometric measurements – only including boys (PDF 19 kb) [file 12887_2019_1500_MOESM3_ESM.pdf]

Supplementary Figure 3.

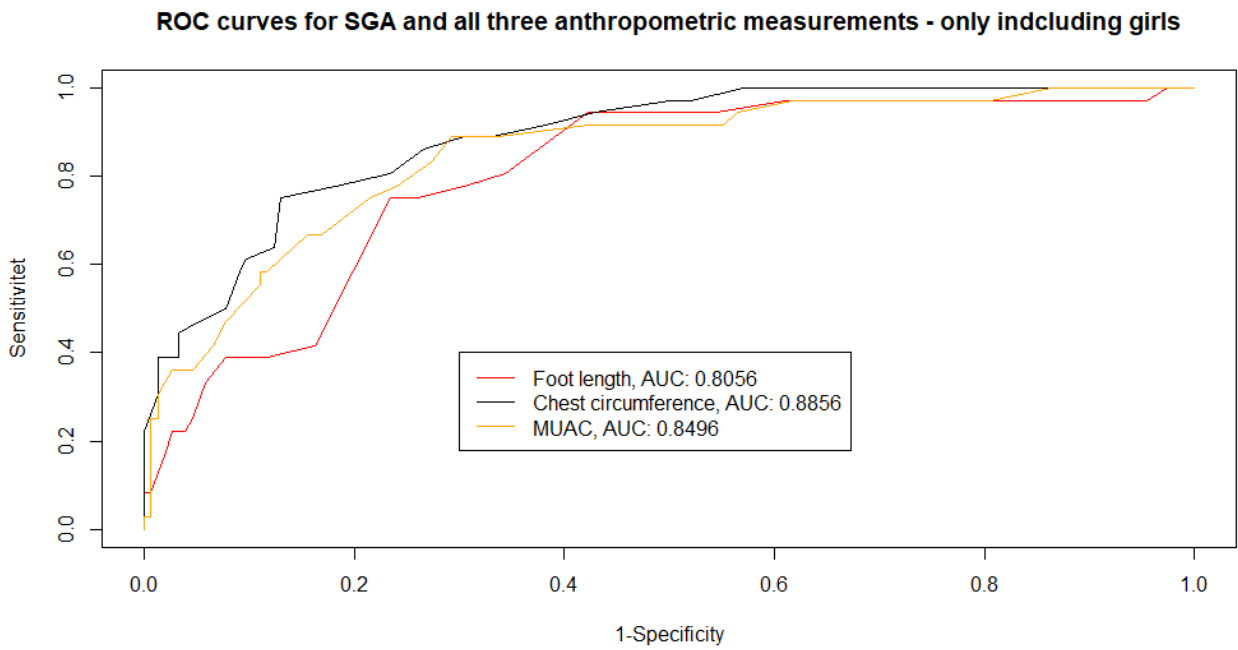

Supplement: Supplementary file 4 — Figure S3. ROC curves for SGA and all three anthropometric measurements – only including girls (PDF 34 kb) [file 12887_2019_1500_MOESM4_ESM.pdf]
