# Supplementary material for: Anthropometric measurements can identify small for gestational age newborns: a cohort study in rural Tanzania
Source: BMC Pediatr. 2019 Apr 23;19:120. doi: 10.1186/s12887-019-1500-0 (PMC6477730; doi:10.1186/s12887-019-1500-0)
Supplement: Supplementary file 5 — Table S2. Foot length cut-off identifying small for gestational age when stratified by sex together with the 95% confidence interval (CI) (PDF 57 kb) [file 12887_2019_1500_MOESM5_ESM.pdf]

Supplementary Table 2. Foot length cut-off identifying SGA[1] when stratified by sex together with the 95% confidence interval (CI).

	Cut-off (cm)	Sensitivity (%) (95% CI)	Specificity (%) (95% CI)
Boys	Foot length ≤ 7.7	66 (47 – 81)	72 (64 – 79)
Girls	Foot length ≤ 7.6	75 (58 – 88)	74 (66 – 81)

[1] SGA = Small for gestational age
